# Florzolotau Tau PET for the prediction of clinical progression in the Shanghai Memory Study

**DOI:** 10.1002/alz70856_102450

**Published:** 2025-12-25

**Authors:** Jiaying Lu, Jie Wang, Huiwei Zhang, Jie Wu, Huamei Lin, Xiaoxi Ma, Ming Li, Zhenxu Xiao, Zizhao Ju, Xiaowen Zhou, Weiqi Bao, Ding Ding, Tzu‐Chen Yen, Chuantao Zuo, Yihui Guan, Qianhua Zhao, the Shanghai Memory Study

**Affiliations:** ^1^ Department of Nuclear Medicine and PET Center, Huashan Hospital, Fudan University, Shanghai, Shanghai, China; ^2^ Institute and department of Neurology, Huashan Hospital, Fudan University, Shanghai, China; ^3^ Department of Nuclear Medicine and PET Center, Huashan Hospital, Fudan University, Shanghai, China; ^4^ National Clinical Research Center for Aging and Medicine, Huashan Hospital, Fudan University, Shanghai, China; ^5^ Institute of Neurology, Huashan Hospital, Fudan University, Shanghai, China; ^6^ National Center for Neurological Disorders, Huashan Hospital, Fudan University, Shanghai, China; ^7^ APRINOIA Therapeutics Co. Ltd, Suzhou, China; ^8^ Human Phenome Institute, Fudan University, Shanghai, China; ^9^ Huashan Hospital, Fudan University, Shanghai, Shanghai, China; ^10^ MOE Frontiers Center for Brain Science, Fudan University, Shanghai, China

## Abstract

**Background:**

Clinical‐biological diagnosis of Alzheimer's disease (AD) is gaining increasing recognition. Beyond diagnosis, biological profiles can be of great benefit for disease monitoring and prognosis, although evidence from real‐world memory clinics is still limited.

**Method:**

211 subjects with cognitive concerns and 31 cognitive unimpaired (CU) volunteers visiting our memory clinic with longitudinal follow‐up were enrolled (mean (SD) interval: 1.89 (1.19) years). All participants received AD pathological evaluations (core 1: amyloid‐PET or plasma *p*‐tau 217; core 2: Florzolotau tau‐PET) at baseline. Biomarkers were assessed by binary amyloid and tau status, and quantitative tau burden in key regions (medial temporal lobe (MTL), neocortical areas (NEO)). Clinical progression served as the primary outcome (increase in CDR or annual MMSE decline ≥ 6). The prognostic values of biomarkers were compared with basic demographic characteristics (a combination of significant risks in Cox Proportional Hazard Models) by receiver operating characteristic analysis.

**Result:**

91 (37.6%) participants were classified as clinical progression (0 CU (0%), 0 SCD (0%), 41 MCI (31.5%) and 50 (56.8%) AD dementia). Only age (older, HR = 1.03, 95%CI 1.01‐1.07, *P* = 0.005) and sex (female, HR = 1.61, 95%CI 1.02‐2.52, *P* = 0.039) showed the significant risks for clinical progression. In the entire cohort, incorporating any of the above biomarkers individually into the basic model (AUC = 0.57) significantly improved the prognostic value (AUC = 0.73‐0.80, all *P* < 0.001), with quantitative tau burden in NEO showing the best added value. Combining both amyloid and tau biomarkers into the basic model further slightly improved the prognosis (AUC = 0.77‐0.82), with the one with binary amyloid status plus quantitative tau burden in NEO showing the best added value. In the A+ subcohort, prognostic value was significantly increased by incorporating the quantitative tau burden (MTL: AUC = 0.71, *P* < 0.001; NEO: AUC = 0.73, *P* < 0.001) into the basic model (AUC = 0.50), while binary tau status didn’t achieve significant improvement (AUC = 0.60, *P* = 0.125).

**Conclusion:**

AD core biomarkers are promising in improving clinical prognosis, among which quantitative tau burden is preferable to binary assessment, especially in the AD continuum.

